# Smoking Cessation and Changes in Anxiety and Depression in Adults With and Without Psychiatric Disorders

**DOI:** 10.1001/jamanetworkopen.2023.16111

**Published:** 2023-05-31

**Authors:** Angela Difeng Wu, Min Gao, Paul Aveyard, Gemma Taylor

**Affiliations:** 1 Nuffield Department of Primary Care Health Sciences, University of Oxford, Oxford, United Kingdom; 2 Addiction and Mental Health Group, Department of Psychology, University of Bath, Bath, United Kingdom

## Abstract

**Importance:**

Although many people report a desire to quit smoking, concerns about mental health worsening after quitting are often raised by clinicians and people who smoke.

**Objective:**

To assess changes in mental health following smoking cessation using 3 confirmatory coprimary analytical approaches.

**Design, Setting, and Participants:**

This cohort study was conducted using data from a large, randomized clinical trial, the Evaluating Adverse Events in a Global Smoking Cessation Study. Analytical approaches included multivariable Tobit regression, propensity score adjustment, and instrumental variable regressions conducted from August to October 2022. Missing data were imputed for sensitivity analysis. The trial occurred in 16 countries at 140 centers between 2011 and 2015. Only data from participants who completed the trial collected in the US were available for this secondary analysis. Participants included adults with or without a psychiatric disorder who smoked.

**Exposure:**

Smoking abstinence between weeks 9 through 24.

**Main Outcomes and Measures:**

Anxiety and depression scores were measured using the Hospital Anxiety and Depression Scale at 24 weeks, where a lower score indicates better mental health (range, 0-21).

**Results:**

Of the 4260 participants included (mean [SD] age, 46.5 [12.4] years; 2485 women [58.3%]; 3044 White individuals [71.5%]), 2359 (55.4%) had a history of mental illness. The mean (SD) baseline Hospital Anxiety and Depression Scale score was 4.25 (3.68) (median [IQR], 3 [1-6]) for anxiety and 2.44 (2.91) (median [IQR], 1 [0-4]) for depression. After adjustment for demographics and baseline variables, smoking cessation was associated with a decrease in scores for both anxiety (−0.40 point; 95% CI, −0.58 to −0.22 point) and depression (−0.47 point; 95% CI, −0.61 to −0.33 point) compared with continuing smoking. Similarly, propensity score–adjusted models indicated that smoking cessation was associated with reduced scores for anxiety (β = −0.32; 95% CI, −0.53 to −0.11) and depression (β = −0.42; 95% CI, −0.60 to −0.24). Instrumental variable analysis was underpowered, and estimates were imprecise. Findings were robust to planned sensitivity and subgroup analyses, with larger effect sizes in people with a history of mental illness.

**Conclusions and Relevance:**

In this cohort study of people with and without psychiatric disorders, smoking cessation, sustained for at least 15 weeks, was associated with improved mental health outcomes in observational analyses, but the instrumental variable analysis provided inconclusive evidence. Findings like these may reassure people who smoke and their clinicians that smoking cessation likely will not worsen and may improve mental health.

## Key Points

**Question:**

Is smoking cessation associated with changes in anxiety and depression for adults living with and without psychiatric disorders?

**Findings:**

In this cohort study of 4260 adults, smoking cessation was associated with significant improvements in anxiety and depression among people with and without psychiatric disorders.

**Meaning:**

These findings suggest that smoking cessation does not appear to worsen and may improve mental health outcomes.

## Teaser

This cohort study assesses changes in anxiety and depression following smoking cessation using 3 confirmatory coprimary analytical approaches.

## Introduction

Many people who smoke state that they want to quit,^1^ but many continue because they report that smoking helps relieve stress and offers other mental health benefits.^2,3,4,5,6,7,8^ These apparent benefits may be spurious. Feelings of low mood, irritability, and anxiety can manifest shortly after finishing a cigarette when blood levels of nicotine drop,^9,10^ and these feelings are relieved by smoking another cigarette.^11,12,13^ Therefore, individuals may perceive that smoking relieves their psychological distress; however, this distress may have been caused by smoking withdrawal. The belief that cigarettes are calming is widespread, and some health professionals may deter people with mental health disorders from trying to stop smoking.^14,15^

A recent Cochrane systematic review^16^ of observational data found that smoking cessation was associated with improved mental health symptoms compared with continuing to smoke for anxiety symptoms (standardized mean difference [SMD], −0.28; 95% CI, −0.43 to −0.13), depression symptoms (SMD, −0.30; 95% CI, −0.39 to −0.21), and mixed anxiety and depression symptoms (SMD, −0.31; 95% CI, −0.40 to −0.22). The magnitude of association was similar in people with or without psychiatric disorders.^17^ Observational studies have found that people who stop smoking are less likely to be prescribed antidepressants and anxiolytics.^18^ However, observational data are at risk of confounding; therefore, the associations reported between smoking cessation and improved mental health outcomes should be taken with caution.

Randomly assigning people to continue or quit smoking is not feasible; therefore, observational methods are the only way to study the association between smoking cessation and mental health. However, traditional observational epidemiology has a primary issue: determining whether associations are causal. One concern is reverse causality (ie, improved mental health may make successful quitting more likely). A systematic review^19^ found evidence that people with psychiatric disorders were less likely to achieve abstinence in any given quit attempt. A second concern is confounding; many studies contributing to the aforementioned meta-analysis^19^ were not adjusted for potential confounding. Poor mental health is associated with factors such as socioeconomic status, which is associated with a lower likelihood of smoking cessation. Thus, existing studies have limitations that decrease the confidence that the association is causal. Given that the rate of smoking in people with diagnosed psychiatric disorders is not decreasing as quickly as in the general population,^18,20^ it is essential to assess whether cessation affects mental health, particularly in people with psychiatric disorders.

This study used 3 confirmatory coprimary analyses—multivariable regression models, propensity score–adjusted models, and instrumental variable (IV) regressions—to evaluate the association between smoking cessation and mental health outcomes. The multivariable regression model will have the least control for confounding, propensity score–adjusted modeling will have less susceptibility,^21^ and confounding factors are unlikely to impact IV analysis. Comparing these analyses facilitates stronger confidence in answering questions regarding the validity of associations if all 3 statistical approaches point to the same conclusion.^22^ Therefore, we attempted to overcome the weaknesses of traditional observational epidemiology and use these methods to evaluate the association of smoking cessation with mental health outcomes.

## Methods

This cohort study followed Strengthening the Reporting of Observational Studies in Epidemiology (STROBE) reporting guidelines for observational studies.^23^ The analytical code can be accessed via GitHub,^24^ and the study protocol is available online.^25^

### Study Design

We used a longitudinal cohort design with individual-level patient data from a double-blind, randomized, placebo-controlled clinical trial of varenicline compared with nicotine replacement therapy, bupropion, or placebo for smoking cessation in people selected because they had or did not have psychiatric disorders (Evaluating Adverse Events in a Global Smoking Cessation Study [EAGLES]).^26^ We compared the change in mental health outcomes from baseline to follow-up (outcome) in those who quit smoking with those who continued to smoke (exposure) rather than by treatment allocation.

### Data Source

Pfizer and GlaxoSmithKline jointly conducted and funded the EAGLE trials,^26^ which were conducted at 140 centers across 16 countries between 2011 and 2015, with 8144 participants enrolled. Only data collected in the US were available for this analysis. The trial was approved by the institutional review boards or ethics committees at participating institutions, with participants providing informed consent to additional anonymized analyses.

### Participants

People aged 18 to 75 years who smoked 10 or more cigarettes per day during the previous year and were motivated to quit were included. Participants were assessed using the Structured Clinical Interview for the *Diagnostic and Statistical Manual of Mental Disorders* (Fourth Edition, Text Revision) diagnostic criteria for psychiatric disorders, including primary mood disorders, anxiety disorders, and psychotic disorders, with no exacerbations of their condition in the preceding 6 months. Those with co-occurring alcohol or substance abuse and dependence were eligible if they had been in full remission for at least 12 months before the trial. More information on the inclusion and exclusion criteria and the informed consent process can be found in the original trial.^26^

### Exposure

Smoking cessation was defined as continuous abstinence between weeks 9 and 24. There were no missing exposure data.

### Outcome

Anxiety and depression outcomes were measured using the Hospital Anxiety and Depression Scale (HADS).^27^ This scale was administered at baseline and through weeks 6, 8, 10, 12, 13, 16, 20, 21, 22, 23, and 24. The HADS consists of 14 individual item responses ranging in increasing severity from 0 (normal) to 3 (most severe). Seven items assess anxiety, and 7 assess depression, providing 2 subscales with ranges of 0 to 21, with a lower score representing lower intensity of symptoms. The outcome was depression and anxiety measured by the HADS at week 24.

### Covariates

Covariates included patients’ age, sex, race, receipt of cessation medication, history of cardiovascular disease (CVD), history of diabetes, psychiatric history, nicotine dependence score, prescription of psychotropics, and body mass index (calculated as weight in kilograms divided by height in meters squared). The method and rationale of racial or ethnic breakdown was not given in the trial report.^26^ Nicotine dependence score was measured using the Fagerstrom Test for Nicotine Dependence, where higher scores indicate greater dependence.^28^

### Statistical Analysis

Analyses were conducted using Stata statistical software version 16 (StataCorp) from August to October 2022. We ran 3 main models—multivariable Tobit regression, propensity score matching (PSM), and IV analysis. Owing to regression to the mean when using within-person change scores, we used the 24-week follow-up HADS scores, with adjustment for baseline HADS score.^29^ All models were adjusted for the aforementioned covariates.

#### Multivariable Tobit Regression

Tobit regression was used because nearly one-third of participants had HADS scores of 0 for depression and almost one-fifth had scores of 0 for anxiety at baseline, making linear regression unfeasible (details are shown in eAppendix 1 in Supplement 1). To investigate the association of continuous smoking abstinence from weeks 9 to 24 on depression and anxiety, we conducted a multivariable-adjusted Tobit regression, with results reported as β coefficients and 95% CIs in HADS score, where β represents the amount of change in the outcome as a function of stopping smoking.

#### Propensity Score Matching

Each participant’s propensity score represents their odds of belonging to the exposure group according to baseline characteristics derived from a logistic regression.^30,31,32^ We matched individuals who quit smoking to people who continued smoking with the closest propensity score on a ratio of 1:1 using the nearest neighbor algorithm with no replacement, restricting matching to the common support region.^33^ We conducted related standard model adequacy checks to examine the distribution of baseline characteristics between matched and unmatched groups. We also ran a post hoc PSM sensitivity analysis by 1:6 matching of people who quit smoking with those who continued (eAppendix 2 and eAppendix 3 in Supplement 1).

#### IV Analysis Procedure

We ran a 2-stage IV regression. Randomization to placebo or active drug was the instrument, which was associated with increased abstinence in the EAGLES trial.^26^ IV analysis was conducted using the ivregress command in Stata. We used the Cragg-Donald Wald *F* statistic and the Hausman test for endogeneity to test for weak instrument bias.^34,35^ We analyzed the power post hoc.^36^

#### Missing Baseline Covariate Data

To increase efficiency and minimize selection bias, we used multivariable multiple imputation to impute data for patients missing values for CVD (4 patients), diabetes (3 patients), and body mass index (39 patients). The imputation procedure produced 20 imputed data sets combined using Rubin rules,^37^ and the imputation model included all exposures and covariates.

#### Sensitivity and Subgroup Analysis

People with a missing HADS score at week 24 may have had worse mental health outcomes. Therefore, running only a complete case analysis may have underestimated change in HADS scores. To examine this, we conducted a sensitivity analysis in which we imputed missing outcome data using multivariate multiple imputation.^37^ The imputation procedure produced 20 imputed data sets, and the imputation model included all exposures and covariates.^38^ We compared the effect estimates derived from the sensitivity analysis (missing outcome data were imputed) with those derived from the primary analysis of complete cases (missing outcome data were dropped from analysis). Bupropion was randomly assigned to 1067 participants in our study. Bupropion is an antidepressant and could be independently associated with mood.^39^ Therefore, we reran the analysis excluding people randomized to receive bupropion. We conducted a subgroup analysis split by participants’ history of psychiatric disorders using the same adjusted Tobit regression model.

## Results

### Participants

We included 4260 participants (mean [SD] age, 46.5 [12.4] years; 2485 women [58.3%]; 3044 White individuals [71.5%]); 2359 (55.4%) had a history of mental illness, and 923 (21.7%) were currently prescribed psychotropic medication (Figure and Table 1). All had smoking status recorded at baseline and follow-up. One individual had a missing record for race; therefore, we recoded the individual as other. People who continued smoking were significantly more likely than those who stopped smoking to be younger, to use psychotropic medication, and to have a higher nicotine dependence score.

**Figure.**
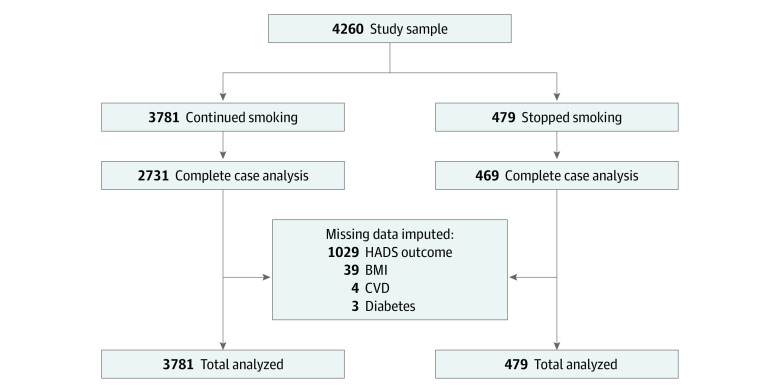
Flow Diagram of Participants Included in Analysis BMI indicates body mass index; CVD, cardiovascular disease; and HADS, Hospital Anxiety and Depression Scale.

**Table 1.** Baseline Characteristics by Smoking Status at 24-Week Follow-up

Characteristic	Participants, No. (%)		
	Whole cohort (N = 4260)	Continued smoking (n = 3781)	Stopped smoking (n = 479)
Age, y			
18-30	573 (13.5)	528 (14.0)	45 (9.4)
31-40	752 (17.7)	677 (17.9)	75 (15.7)
41-50	1129 (26.5)	1008 (26.7)	121 (25.3)
51-60	1244 (29.2)	1096 (29.0)	148 (30.9)
61-75	562 (13.2)	472 (12.5)	90 (18.8)
Sex			
Male	1775 (41.7)	1577 (41.7)	198 (41.3)
Female	2485 (58.3)	2204 (58.3)	281 (58.7)
Race			
White	3044 (71.5)	2662 (70.4)	382 (79.7)
Black	1065 (25.0)	990 (26.2)	75 (15.7)
Asian	32 (0.8)	27 (0.7)	5 (1.0)
Other^a^	119 (2.8)	102 (2.7)	17 (3.5)
Body mass index group^b^			
<18.5	63 (1.5)	58 (1.5)	5 (1.0)
18.5 to <25	1054 (24.7)	951 (25.2)	103 (21.5)
25 to <30	1373 (32.2)	1203 (31.8)	170 (35.5)
>30	1731 (40.6)	1535 (40.6)	196 (40.9)
Psychiatric history	2359 (55.4)	2106 (55.7)	253 (52.8)
Current use of psychotropic medication	923 (21.7)	852 (22.5)	71 (14.8)
Diabetes	300 (7.0)	264 (7.0)	36 (7.5)
Evidence of cardiovascular disease	201 (4.7)	180 (4.8)	21 (4.4)
Randomized treatment group			
Bupropion	1067 (25.0)	945 (25.0)	122 (25.5)
Nicotine replacement therapy	1062 (24.9)	949 (25.1)	113 (23.6)
Placebo	1065 (25.0)	993 (26.3)	72 (15.0)
Varenicline	1066 (25.0)	894 (23.6)	172 (35.9)
Baseline scores, mean (SD)			
Nicotine dependence	5.71 (1.93)	5.77 (1.91)	5.23 (2.02)
Depression	2.44 (2.91)	2.47 (2.93)	2.22 (2.80)
Anxiety	4.25 (3.68)	4.30 (3.61)	3.85 (3.44)

^a^ Information on breakdown of subcategories included is unavailable.

^b^ Body mass index is calculated as weight in kilograms divided by height in meters squared.

### Outcome Data

There were 1224 participants (28.7%) with missing HADS scores for week 24. We replaced missing HADS score with the last score recorded after 20 weeks, leaving 1029 participants (24.2%) who had missing outcome data on HADS and were excluded from the complete case analysis. The mean (SD) baseline HADS score was 4.25 (3.68) (median [IQR], 3 [1-6]) for anxiety and 2.44 (2.91) (median [IQR], 1 [0-4]) for depression.

### Complete Case Analysis

We included 3200 people; 2731 continued and 469 stopped smoking. Mental health scores improved in both groups (Table 2 and Table 3).

**Table 2.** Hospital Anxiety and Depression Scale Scores for Participants by Smoking Status and Unadjusted Complete Case Tobit Regression

Outcome and time	Score, mean (SD)
Anxiety^a^	
Continued smoking	
Baseline	4.31 (3.67)
Follow-up	2.29 (3.43)
Stopped smoking	
Baseline	3.81 (3.43)
Follow-up	1.51 (2.79)
Depression^b^	
Continued smoking	
Baseline	2.51 (2.92)
Follow-up	1.67 (2.77)
Stopped smoking	
Baseline	2.21 (2.81)
Follow-up	0.92 (2.10)

^a^ β = −0.28 (95% CI, −0.41 to −0.14).

^b^ β = −0.29 (95% CI, −0.4 to −0.19).

**Table 3.** Change in Hospital Depression and Anxiety Scale Scores for People Who Remained Abstinent Between Weeks 9 and 24

Outcome	β (95% CI)
Depression	
Tobit complete adjusted	−0.47 (−0.61 to −0.33)
PSM Tobit adjusted	−0.42 (−0.6 to −0.24)
Tobit removal of bupropion	−0.37 (−0.53 to −0.21)
Tobit without psychiatric history	−0.32 (−0.48 to −0.15)
Tobit with psychiatric history	−0.60 (−0.82 to −0.38)
Multiple imputation Tobit	−0.97 (−1.15 to −0.78)
Multiple imputation PSM	−0.53 (−0.67 to −0.4)
Anxiety	
Tobit complete adjusted	−0.40 (−0.58 to −0.22)
PSM Tobit adjusted	−0.32 (−0.53 to −0.11)
Tobit removal of bupropion	−0.33 (−0.53 to −0.12)
Tobit without psychiatric history	−0.29 (−0.50 to −0.80)
Tobit with psychiatric history	−0.48 (−0.76 to −0.20)
Multiple imputation Tobit	−0.33 (−0.53 to −0.12)
Multiple imputation PSM	−0.54 (−0.7 to −0.37)

Abbreviation: PSM, propensity score matching.

### Unadjusted and Multivariable Adjusted Tobit Regression

People who stopped smoking had a lower HADS score at week 24 than those who continued smoking. Anxiety was lower by −0.28 point (95% CI, −0.41 −0.14 point) unadjusted and −0.40 point (95% CI, −0.58 to −0.22 point) adjusted. Depression was lower by −0.29 point (95% CI, −0.40 to −0.19 point) unadjusted and −0.47 point (95% CI, −0.61 to −0.33 point) adjusted. There was no evidence of a difference between people who were randomized to active drugs vs those randomized to placebo in mental health outcomes (eTable 1 and eTable 2 in Supplement 1).

### PSM Results

The difference in bias was reduced after PSM, and the common support region was large (eAppendix 3 and eFigure in Supplement 1). After matching, both people who stopped and continued smoking experienced improved mental health (Table 3). The difference between groups was β = −0.42 (95% CI, −0.60 to −0.24) for depression and β = −0.32 (95% CI, −0.53 to −0.11) for anxiety, indicating that stopping smoking was associated with improved mental health. PSM with 1:6 ratio produced very similar results (eAppendix 2 in Supplement 1).

### IV Analysis

IV analysis suggested that depression increased relative to continuing smoking (β = 1.48; 95% CI, −0.67 to 3.63), as did anxiety (β = 1.60; 95% CI, −1.01 to 4.22), but both estimates were imprecise. Post hoc power analysis suggested that we had very little power to exclude effect sizes like those seen in our observational analyses (eAppendix 4 in Supplement 1).

### Sensitivity and Subgroup Analysis

Sensitivity analysis removing individuals randomized to bupropion did not materially change the estimates compared with the complete case Tobit model: β = −0.33 (95% CI, −0.53 to −0.12) for anxiety and β = −0.37 (95% CI, −0.53 to −0.21) for depression. Following multiple imputation, there was a difference in adjusted anxiety scores of β = −0.33 (95% CI, −0.53 to −0.12) for the Tobit model and β = −0.54 (95% CI, −0.70 to −0.37) for PSM and adjusted depression scores of −0.97 point (95% CI, −1.15 to −0.78 point) for the Tobit model and −0.53 point (95% CI, −0.67 to −0.40 point) for PSM.

Among individuals without a psychiatric history who stopped smoking, the decreases in depression (−0.32 point; 95% CI, −0.48 to −0.15 point) and anxiety (−0.29 point; 95% CI, −0.50 to −0.08 point) scores were smaller than the decreases observed among individuals with a psychiatric history who stopped smoking (depression, −0.60 point; 95% CI, −0.82 to −0.38 point; anxiety, 0.48 point; 95% CI, −0.76 to −0.20 point).

## Discussion

### Main Findings

In this cohort study, people who stopped smoking showed lower depression and anxiety scores 6 months after stopping smoking than people who continued smoking after adjustment for a range of possible confounding variables or with PSM. Scores were 0.40 point lower for anxiety and 0.47 point lower for depression on the HADS scale. Sensitivity analysis removing people randomly assigned to bupropion left the results unchanged. Sensitivity analyses using multiple imputation showed that effect estimates were in a similar direction of association, but point estimates were somewhat larger than in the complete case analyses. The association between smoking cessation and improvements in mental health was larger when we restricted it to those with a history of mental illness. Our IV analysis was inconclusive but lacked the power to detect an effect of smoking cessation on mental health.

### Interpretation

Our analyses suggest that smoking cessation is associated with improved mental health. However, our IV analysis was unable to confirm this.^40^ The disparity between smoking cessation rates in people with and without psychiatric disorders is concerning, given that smoking may account for up to two-thirds of the difference in life expectancy between people with a history of psychiatric disorders who smoke vs people who have never smoked.^41^ Smoking cessation is associated with decreased morbidity risk and improved quality of life at any age,^42^ with our analysis adding to evidence that it improves mental well-being too. Smoking is the leading cause of preventable disease and death in the world,^43^ with 1 in every 2 people who continue smoking through life dying from a smoking-related disease.^44^ In many countries, such as the United Kingdom, the prevalence of smoking in the general population has decreased from approximately 46% in the 1970s to 13.3% in 2021.^45^ The decrease in prevalence has not been as large among people with mental health conditions, with the prevalence remaining at approximately 40% from 1993 to 2013.^46^ The EAGLES trial showed that cessation medication was not associated with adverse neuropsychiatric effects.^47^

### Limitations

Although the instrument of the randomized treatment group is theoretically sound, the instrument was not robust in this data set. Given the low likelihood of detecting an association of the size we observed in the observational data with the IV analysis with our sample size, future work would need a larger sample size. Our results, therefore, do not exclude a causal relationship between smoking cessation and improved mental health for people with and without psychiatric disorders.

This study had substantial loss to follow-up, which is common in smoking cessation trials. Evidence suggests that those who fail to attend follow-up are more likely to continue smoking.^48^ Assuming scores were missing at random with multiple imputation, the association of cessation with decreased anxiety and depression was larger than that seen in the complete case analysis. It seems likely that imputing these missing HADS scores as typical of those who continue smoking would also increase not decrease the apparent difference between groups. However, the true reasons for missing data at follow-up cannot be known from secondary data analysis. Data on participant socioeconomic status were not collected, so it was not possible to adjust for socioeconomic status. However, socioeconomic status is a time-invariant variable and is unlikely to confound changes in mental health.

The EAGLES trial^26^ was conducted in 16 countries; however, the data sharing agreement allowed us access only to data from trial sites in the US. This may limit generalizability, but if the association between cessation and mood change is related to neurobiological changes consequent on ceasing regular nicotine use, then these data are likely to generalize to other countries and populations.

Like many other smoking cessation studies, this study classified people who did not meet the strict definition of abstinence as continuing smoking. Those people who had achieved abstinence but not had 15 weeks of abstinence may have experienced changes in their mental health similar to those who met the 15-week abstinence, as the evidence suggests that the association is of similar strength in people with only 6 weeks of abstinence after several years.^16^ In our study, such participants would have been counted as continuing smoking, which may dilute our observed association size.

Although participants with and without a psychiatric history showed reduced HADS scores, these were less than the minimally clinically important differences suggested by populations with chronic obstructive pulmonary disease and CVD.^49,50^ The reduction was larger for those with a psychiatric history, perhaps because they had higher baseline scores, allowing for a larger decrease to be manifest. Participants were included only if they were not currently experiencing a mental illness, so the results may not generalize to those with current illness. Nonetheless, the neurobiological changes associated with quitting smoking are likely to apply to all individuals, regardless of any underlying condition. People with a high risk of suicide were not included in the trial, but it is unlikely that those individuals would attempt to stop smoking. Although the trial excluded people at high risk of suicide because it was a concern with varenicline prescription, the EAGLES trial^26^ showed no increased risk of adverse psychiatric events with varenicline, and other studies show either inconclusive evidence^51^ or no evidence of increased risk of suicide associated with varenicline.^52,53^

The EAGLES trial^26^ was the largest trial of smoking cessation medication that we know, and yet our IV analysis lacked power to detect an effect of smoking cessation on mental health outcomes. As such, it is unlikely that this type of analysis will be possible in the future without pooling data. That said, most of the trials included in a recent systematic review^16^ on the association between cessation and mental health tested interventions that do not improve cessation and thus cannot be valid instruments. As such, it may be impossible to prove causality using an IV approach at present.

## Conclusions

In this cohort study of people with and without psychiatric disorders, we found that smoking cessation was associated with improved mental health outcomes. These findings might motivate policy makers and stakeholders to support smoking cessation in people with mental health disorders.

## Notes

Supplement 1. **eAppendix 1.** Tobit Regression **eTable 1.** Adjusted Tobit Regression Model for Outcome of Depression **eTable 2.** Adjusted Tobit Regression Model for Outcome of Anxiety **eAppendix 2.** PSM **eFigure.** Kernel Density Estimate Plot of the Probability Distribution of Propensity Scores Before and After Matching **eAppendix 3.** Sensitivity Analysis **eAppendix 4.** IV Analysis
eReferences

Supplement 2.
Data Sharing Statement
