# Supplementary material for: Smoking Cessation and Changes in Anxiety and Depression in Adults With and Without Psychiatric Disorders
Source: JAMA Netw Open. 2023 May 31;6(5):e2316111. doi: 10.1001/jamanetworkopen.2023.16111 (PMC10233414; doi:10.1001/jamanetworkopen.2023.16111)
Supplement: Supplement 1. — eAppendix 1. Tobit Regression eTable 1. Adjusted Tobit Regression Model for Outcome of Depression eTable 2. Adjusted Tobit Regression Model for Outcome of Anxiety eAppendix 2. PSM eFigure. Kernel Density Estimate Plot of the Probability Distribution of Propensity Scores Before and After Matching eAppendix 3. Sensitivity Analysis eAppendix 4. IV Analysis eReferences [file jamanetwopen-e2316111-s001.pdf]

Supplemental Online Content

Wu AD, Gao M, Aveyard P, Taylor G. Smoking cessation and changes in anxiety and depression in adults with and without psychiatric disorders. *JAMA Netw Open*. 2023;6(5):e2316111. doi:10.1001/jamanetworkopen.2023.16111

eAppendix 1. Tobit Regression
eTable 1. Adjusted Tobit Regression Model for Outcome of Depression
eTable 2. Adjusted Tobit Regression Model for Outcome of Anxiety
eAppendix 2. PSM
eFigure. Kernel Density Estimate Plot of the Probability Distribution of Propensity Scores Before and After Matching
eAppendix 3. Sensitivity Analysis
eAppendix 4. IV Analysis
eReferences

This supplemental material has been provided by the authors to give readers additional information about their work.

© 2023 Wu AD et al. *JAMA Network Open.*

eAppendix 1. Tobit Regression

We selected the Tobit regression model because the distribution of HADS scores was not normal but skewed and censored at 0 and 21 (range of the scores 0-21). Secondly, a substantial proportion (16% for anxiety and 31% for depression) of the observations was at the lower limit of 0 at baseline.

eTable 1. Adjusted Tobit Regression Model for Outcome of Depression

**Predictors**	**coefficient**	**p value**	**95% CI Lower**	**95% CI Upper**
Smoking Abstinence	-0.47	p<0	-0.61	-0.33
HADS Depression Baseline	0.31	p<0	0.29	0.33
Active Drug	0.11	0.07	-0.01	0.23
Age Group = ref Aged 18/30				
31/40	0.05	0.64	-0.15	0.25
41/50	0.21	p<0.05	0.03	0.4
51/60	0.39	p<0	0.21	0.57
61/75	0.52	p<0	0.31	0.74
Female	0.09	0.12	-0.02	0.2
Race = ref White				
Black	-0.07	0.27	-0.19	0.53
Asian	0.69	0.05	0	0.37
Other	0.17	0.35	-0.18	0.51
BMI = ref BMI less than18.5				
18.5 to less than 25	0.25	0.28	-0.2	0.7
25 to less than 30	0.28	0.22	-0.17	0.73
Greater than 30	0.15	0.5	-0.29	0.6
Nicotine Dependence	0.01	0.42	-0.02	0.4
Psychiatric History	0.35	p<0	0.23	0.46
Psychotropic Medication	0.22	p<0.001	0.09	0.36
Cardiovascular Disease	0.09	0.46	-0.16	0.34
Diabetes	-0.06	0.56	-0.25	0.13

eTable 2. Adjusted Tobit Regression Model for Outcome of Anxiety

**Predictors**	**coefficient**	**p value**	**95% CI Lower**	**95% CI Upper**
Smoking Abstinence	-0.4	p<0	-0.58	-0.22
HADS Anxiety Baseline	0.35	p<0	0.33	0.37
Active Drug	0.06	0.46	-0.09	0.21
Age Group = ref Aged 18/30				
31/40	0.16	0.2	-0.08	0.41
41/50	0.32	p<0.01	0.09	0.54
51/60	0.49	p<0	0.26	0.71
61/75	0.69	p<0	0.43	0.96
Female	0.09	0.22	-0.05	0.22
Race = ref White				

© 2023 Wu AD et al. *JAMA Network Open.*

Black	-0.07	0.37	-0.22	0.08
Asian	0.55	0.18	-0.25	0.35
Other	-0.12	0.56	-0.53	0.29
BMI = ref BMI less than18.5				
18.5 to less than 25	0.16	0.59	-0.42	0.73
25 to less than 30	0.13	0.65	-0.44	0.7
Greater than 30	-0.09	0.76	-0.66	0.48
Nicotine Dependence	0.02	0.4	-0.02	0.5
Psychiatric History	0.37	p<0	0.23	0.51
Psychotropic Medication	0.3	p<0	0.13	0.47
Cardiovascular Disease	-0.05	0.76	-0.35	0.25
Diabetes	0.049	0.697	-0.197	0.294

eAppendix 2. PSM

After matching, we checked the balance of means and variances of covariates by examining the standardised mean differences between people who continued to smoke and those who stopped [1]. We calculated the achieved percentage of reduction in bias after matching and examined scatter plots comparing each covariate's standardised per cent bias before and after matching[2]. We also examined the kernel density estimate of the probability distribution of propensity scores before and after matching.

The difference in bias between groups after matching was examined to determine which variables were adequately matched. In all cases, variables significantly imbalanced between groups before matching were no longer significantly imbalanced after matching. Before matching, there were significant differences between the groups' age, race, psychotropic medication, nicotine dependency scores (FTND) and randomised treatment group. There were no cases where variables became significantly imbalanced after matching. In total, all 469 people who stopped smoking were matched to a person who continued; therefore, 938 in total.

As shown in the eFigure, there was a common support area to perform PSM, and participants were predominately matched within the common region.

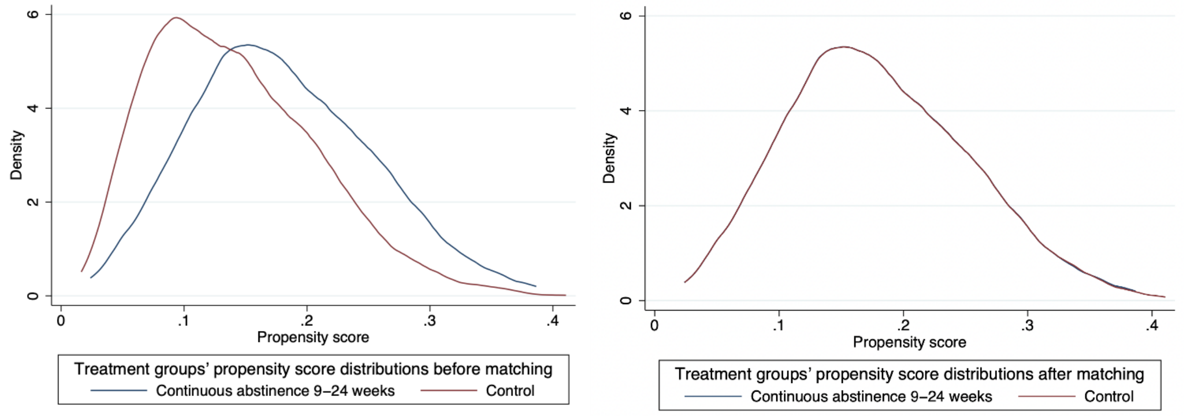

eFigure. Kernel Density Estimate Plot of the Probability Distribution of Propensity Scores Before and After Matching

eAppendix 3. Sensitivity Analysis

We matched individuals who quit smoking to people who continued smoking with the closest propensity score on a ratio of 1:6 using the nearest neighbour algorithm, restricting matching to the common support region. For the outcome of depression all 469 people who stopped smoking were matched to 1564 people, whereas anxiety matched 468 people who stopped to 1560 people who continued. There were no cases where variables became significantly imbalanced after matching. The difference between groups was -0.43 (95% CI [-0.59, -0.28]) for depression and -0.39 (95% CI [-0.58, -0.2]) for anxiety, indicating that stopping smoking was associated with improved mental health.

eAppendix 4. IV Analysis

When analysing the outcome of depression, we rejected the null hypothesis that smoking abstinence was exogenous. However, it was not considered a weak instrument because the F score of the first stage regression was above 10 (score of 33). While the instrument was also considered not weak for the outcome of anxiety, we failed to reject null as the Wu-Hausman score was non-significant. Therefore, we cannot assume endogeneity in the data for the outcome of anxiety. Hence, IV analysis was not appropriate. However, for depression, progression to IV analysis was appropriate as there appeared to be an endogeneity of the data. We ran a post-hoc analysis testing the instrument's power in our given population. Our power calculation demonstrated a likelihood of 14.4% that our analysis will detect a treatment effect of 0.47 (the effect size seen in the observational analysis) using our instrument with a 5% type 1 error rate[3].

© 2023 Wu AD et al. *JAMA Network Open.*

eReferences

1. Thoemmes FJ, Kim ES. A Systematic Review of Propensity Score Methods in the Social Sciences. *Multivariate Behavioral Research*. 2011;46(1):90-118. doi:10.1080/00273171.2011.540475
2. Rosenbaum PR, Rubin DB. Constructing a Control Group Using Multivariate Matched Sampling Methods That Incorporate the Propensity Score. *The American Statistician*. 1985;39(1):33-38. doi:10.2307/2683903
3. Walker VM, Davies NM, Windmeijer F, Burgess S, Martin RM. Power calculator for instrumental variable analysis in pharmacoepidemiology. *International Journal of Epidemiology*. 2017;46(5):1627-1632. doi:10.1093/ije/dyx090

© 2023 Wu AD et al. *JAMA Network Open.*
