# Supplementary material for: Smoking Cessation and Changes in Anxiety and Depression in Adults With and Without Psychiatric Disorders
Source: JAMA Netw Open. 2023 May 31;6(5):e2316111. doi: 10.1001/jamanetworkopen.2023.16111 (PMC10233414; doi:10.1001/jamanetworkopen.2023.16111)
Supplement: Supplement 2. — Data Sharing Statement [file jamanetwopen-e2316111-s002.pdf]

Data Sharing Statement

Wu. Smoking Cessation and Changes in Anxiety and Depression in Adults With and Without Psychiatric Disorders. *JAMA Netw Open*. Published May 31, 2023.
doi:10.1001/jamanetworkopen.2023.16111

Data
Data available: No

Additional Information
Explanation for why data not available: We do now own any of the data analysed in this study, all data was access through a data sharing platform called Vivli
